# The prevalence of sepsis-induced coagulopathy in patients with sepsis – a secondary analysis of two German multicenter randomized controlled trials

**DOI:** 10.1186/s13613-022-01093-7

**Published:** 2023-01-12

**Authors:** Thomas Schmoch, Patrick Möhnle, Markus A. Weigand, Josef Briegel, Michael Bauer, Frank Bloos, Patrick Meybohm, Didier Keh, Markus Löffler, Gunnar Elke, Thorsten Brenner, Holger Bogatsch, for the SepNet–Critical Care Trials Group

**Affiliations:** 1 Department of Anesthesiology and Intensive Care Medicine, Hôpitaux Robert Schuman – Hôpital Kirchberg, 9, Rue Edward Steichen, 2540 Luxembourg City, Luxembourg; 2 Department of Anesthesiology and Intensive Care Medicine, University Hospital Essen, University Duisburg-Essen, Essen, Germany; 3 Department of Transfusion Medicine, Cell Therapeutics and Hemostasis, Department of Anesthesiology, Klinikum Der Ludwig-Maximilians-Universität, Munich, Germany; 4 Department of Anesthesiology, Heidelberg University Hospital, Heidelberg, Germany; 5 Department of Anesthesiology, Klinikum Der Ludwig-Maximilians-Universität, Munich, Germany; 6 Center for Sepsis Control and Care, Jena University Hospital, Jena, Germany; 7 Department of Anesthesiology and Intensive Care Medicine, Jena University Hospital, Jena, Germany; 8 Department of Anesthesiology, Intensive Care, Emergency and Pain Medicine, University Hospital Wuerzburg, Würzburg, Germany; 9 Department of Anesthesiology and Intensive Care Medicine, Charité-Universitätsmedizin Berlin, Berlin, Germany; 10 Institute for Medical Informatics, Statistics and Epidemiology (IMISE) and Clinical Trial Centre Leipzig, Leipzig, Germany; 11 Department of Anesthesiology and Intensive Care Medicine, University Medical Center Schleswig-Holstein, Campus Kiel, Kiel, Germany

**Keywords:** Sepsis, Septic shock, Sepsis-induced coagulopathy, Platelet count, Prevalence

## Abstract

**Background:**

Sepsis and septic shock are frequently accompanied by coagulopathy. Since the sepsis-induced coagulopathy (SIC) score was first described, subsequent studies from Asia revealed a SIC prevalence of 40–60%. In Europe, however, SIC prevalence in patients fulfilling sepsis criteria according to the third international consensus definition (SEPSIS-3) has not yet been evaluated.

**Methods:**

The Critical Care Trials Group of the German Sepsis Competence Network (SepNet) conducted a secondary analysis of two randomized controlled trials. Only patients fulfilling sepsis criteria according SEPSIS-3 were included in this secondary analysis. In a two step approach, SIC prevalence was determined in 267 patients with sepsis but not septic shock (at the time of inclusion) from the “Effect of Hydrocortisone on Development of Shock Among Patients With Severe Sepsis” (HYPRESS) trial. Then, we estimated SIC prevalence in 1,018 patients from the “Effect of Sodium Selenite Administration and Procalcitonin-Guided Therapy on Mortality in Patients With Severe Sepsis or Septic Shock” (SISPCT) trial using a simplified SIC score based on the platelet-SIC-subscore (PSSC). Study aims were to assess (i) the prevalence of SIC in patients with SEPSIS-3, (ii) the association of SIC with 90-day mortality and morbidity, (iii) the time when patients become SIC positive during the course of sepsis, and (iv) the value of the PSSC for predicting SIC.

**Results:**

In the HYPRESS trial, SIC prevalence was 22.1% (95% confidence interval [CI] 17.5–27.5%). The estimated SIC prevalence in the SISPCT trial was 24.2% (95% CI 21.6–26.9%). In the HYPRESS trial, SIC was associated with significantly higher 90-day mortality (13.9% vs. 26.8%, *p* = 0.027) and morbidity. Logistic regression analysis adjusted for age, sex, treatment arm, and (SIC-adapted) SOFA score confirmed the negative association of SIC with survival (*p* = 0.011). In the SISPCT trial, increased PSSCs were associated with higher 90-day mortality (PSSC 0: 34.4%, PSSC 1: 40.5%, PSSC 2: 53.3%; *p* < 0.001). In both trials, SIC was already present at sepsis diagnosis or occurred during the following 4 days.

**Conclusions:**

SIC is a clinically relevant complication of sepsis. Although it might be less frequent than previously reported, its occurrence is associated with higher morbidity and mortality and should be interpreted as an early warning sign.

**Supplementary Information:**

The online version contains supplementary material available at 10.1186/s13613-022-01093-7.

## Background

In patients with sepsis or septic shock, the coagulation system is regularly impaired [1]. Due to the physiological interaction of inflammation and coagulation, disorders of the coagulation system are understood to be an integral part of the “life threatening host response to infection” that defines sepsis [2, 3]. In 2017, the Scientific Standardization Committee on Disseminated Intravascular Coagulopathy (DIC) of the International Society on Thrombosis and Haemostasis (ISTH) established the term sepsis-induced coagulopathy (SIC), introducing a new screening and diagnostic tool called the SIC score [4, 5]. The SIC score provides criteria to diagnose coagulopathy caused by sepsis easier and in an earlier stage [4, 5]. Therefore, in contrast to other DIC-screening tools like the ISTH overt-DIC score [6] and the Japanese Association for Acute Medicine (JAAM) score [7], the SIC score only relies on three components: (1) an adapted Sequential (sepsis-related) Organ Failure Assessment (SOFA) score, (2) the platelet count, and (3) the international normalized ratio (INR; or the prothrombin time [PT]) [4, 8]. Both fibrinogen plasma levels (a component of the ISTH score) and D-dimer plasma levels (a component of both the ISTH and JAAM scores) are no longer integrated (see Additional file 1: Table S1). As a result, the SIC score detects a coagulopathy that does not (yet) necessarily reflect overt DIC. Systemic inflammatory response syndrome (SIRS) criteria, which are part of the JAAM score, were replaced by the SOFA score in order to adapt the SIC score to the Third International Consensus Definitions for Sepsis and Septic Shock (SEPSIS-3) [3]. A SIC score ≥ 4 is considered positive. Moreover, as an additional condition the sum of the Platelet SIC subscore (PSSC) and the INR SIC subscore (ISSC) has to be ≥ 3 [4]. The performance of the SIC score has been validated retrospectively in several Japanese cohorts [5, 9, 10]. However, it remains unclear whether the determined incidences and outcomes are transferable to other, non-Japanese cohorts. The original work through which the SIC score was established used highly preselected patients fulfilling the criteria of severe sepsis and DIC according to the criteria of the Japanese Ministry of Health, Labor and Welfare [4, 11]. Moreover, in contrast to the Surviving Sepsis Campaign (SSC), the Japanese sepsis guidelines recommend the use of antithrombin and thrombomodulin in patients with suspected DIC [12, 13]. As a result, about 50% of the patients included in these validation studies had been treated with at least one of these drugs [5, 10].

Here, we present a secondary analysis of the “Effect of Hydrocortisone on Development of Shock Among Patients With Severe Sepsis: The HYPRESS Randomized Clinical Trial”, and the “Effect of Sodium Selenite Administration and Procalcitonin-Guided Therapy on Mortality in Patients With Severe Sepsis or Septic Shock” (SISPCT) trial [14, 15]. The aims of this analysis were to assess (i) the prevalence of SIC in patients with sepsis (SEPSIS-3), (ii) the association of SIC with mortality and morbidity, (iii) the onset of SIC positivity during the course of sepsis. In a two step approach we first analysed the HYPRESS trial including patients with sepsis but not septic shock (about 22% of these patients developed septic shock during the 14 day observation period) [14]. Then, we analysed the SISPCT trial using a simplified SIC-score based on the SIC-adapted SOFA and PSSC.

## Methods

This study was a secondary analysis of the “The HYPRESS Randomized Clinical Trial” [14, 15] and the SISPCT trial [14, 15]. Only patients fulfilling SEPSIS-3 critera (i.e., infection + SOFA score ≥ 2 points) at the time of inclusion in the original trial were included.

The HYPRESS trial was an investigator-initiated, multicenter, placebo-controlled, double-blind randomized controlled trial (RCT). It was supported by the German Federal Ministry of Education and Research and conducted between January 2009 and February 2014 at 34 intermediate care (IMCs) or intensive care units (ICUs) of university and community hospitals in Germany. A detailed description of the methodology can be found in the primary publication of the trial [14]. Briefly, adult patients with severe sepsis who were not in septic shock (at the time of inclusion) were included and randomized 1:1 either to receive a continuous infusion of 200 mg hydrocortisone (HC) for 5 days followed by dose tapering until day 11 or to receive placebo. The primary outcome, “development of septic shock within 14 days”, was met by 75 of 340 (22.06%) patients [14]. All patients were treated according to the (at that time) valid guidelines of the German Sepsis Society [16]. SIC scores were calculated according to the previously published definition of Iba et al. [4] (see Additional file 1: Table S1). For a SIC score to be considered positive, two conditions had to be met: i) the SIC score had to be  ≥ 4 and ii) the sum of the PSSC and the INR SIC subscore (ISSC) had to be  ≥ 3 [4]. The SIC score uses an adapted SOFA score only taking into account the sum of four subscores, namely respiratory, cardiocirculatory, hepatic, and renal [4, 8].

The SISPCT trial was an investigator-initiated, multicenter RCT performed at 33 ICUs in Germany [15]. The trial included patients with sepsis and septic shock (according to the SEPSIS-2 definition) and was conducted from November 6, 2009, to June 6, 2013, including a 90-day follow-up period [15, 17]. It was designed to investigate the effect of sodium selenite and procalcitonin guidance of anti-infective therapy on the 28-day mortality of patients with sepsis and septic shock. The international normalized ratio (INR) was not recorded during the SISPCT trial. Therefore, SIC prevalence in SISPCT was estimated using a “simplified SIC score”. The simplified SIC score was considerd positive, if the sum of the SIC-adapted SOFA sub score and PSSC was 4.

Before including the first patient, the trial protocols of both trials had been registered (clinicaltrials.gov Identifier: NCT00670254 [18], NCT00832039 [19]) and approved by the leading ethics board of Jena University Hospital and the institutional review boards of all participating institutions [15]. Both trials were carried out according to the Declaration of Helsinki (October 2013) and in both trials, written informed consent, including secondary analyses, was obtained from all study participants [20].

### Statistical analyses

Primary endpoints were SIC prevalence in HYPRESS and the estimated SIC prevalence in SISPCT. Secondary endpoints were the association of SIC with mortality in HYPRESS and SISPCT, as well as the association of SIC with mean SOFA score until day 14, the need of renal replacement therapy (RRT) until day 28 and ICU length of stay (ICU-LOS) in HYPRESS.

Study participant characteristics were compared using the *χ*^2^ test, Fisher’s exact test, the Mann–Whitney U test, the Kruskal–Wallis H test, the Kaplan–Meier estimator, or the log-rank test, as appropriate. Multivariate logistic regression was performed to compare survival of SIC positive and SIC negative patients adjusting for age, sex, treatment arm, and the SIC-adapted SOFA score at sepsis onset (= the time of sepsis diagnosis). The SOFA score was adapted according to Iba et al. [4]. All reported *p* values are two sided. Statistical analyses were performed using SPSS version 28.0 (IBM Corp., Armonk, NY, USA). Data analysis was performed between September 2021 and May 2022. Because the SEPSIS-2 definition was still valid at the time the HYPRESS and the SISPCT trial were conducted, the SOFA score was not part of the mandatory baseline parameters. Likewise, it was not mandatory to provide platelet counts or the INR. If SOFA scores were missing at sepsis onset (the day of diagnosis), they were imputed as the SOFA scores for day 1. If a SOFA score was missing for sepsis onset and day 1, the patient was excluded. If an INR value or platelet count was missing at sepsis onset, the patient was excluded.

## Results

### Study participant characteristics and SIC prevalence in in the HYPRESS trial

**Fig. 1:**
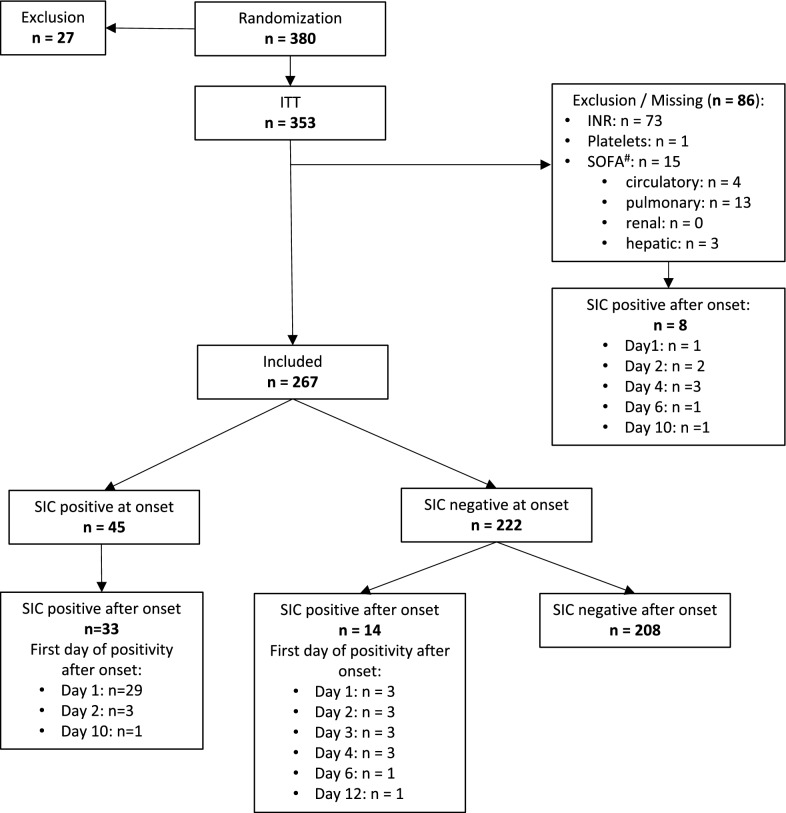
Flow diagram detailing the selection of patient groups analyzed from the HYPRESS trial [14]. ^#^SOFA scores missing at sepsis onset and day 1 (if SOFA subscores were missing at sepsis onset they were imputed as day 1). If the INR or platelet counts were missing at sepsis onset, patients were excluded. *INR* international normalized ratio, *SIC* sepsis-induced coagulopathy; *SOFA* Sequential (sepsis-related) Organ Failure Assessment

From 380 patients that had been randomized in the HYPRESS trial, 27 had to be excluded from the intention-to-treat (ITT) analysis. The remaining 353 patients (ITT) were eligible for our secondary analysis. Eighty-six had to be excluded due to missing sepsis onset data. All remaining 267 patients had “severe sepsis” according to SEPSIS-2 [17] and fulfilled the criteria for “sepsis” according to SEPSIS-3 [3] (Fig. 1). At sepsis onset, 45 (16.9%) patients were SIC positive. During the 14 day observation period, 14 additional patients were SIC positive at least once. Therefore, the SIC prevalence during the observation period was 22.1% (95% confidence interval [CI] 17.5–27.5%).

Patients with and without SIC were comparable regarding the distribution of age and sex (Table 1). However, a positive SIC score at sepsis onset was associated with a higher mean SOFA score at sepsis onset (8 [interquartile range, IQR, 7–10] vs. 5 [IQR 4–7]; Table 1). SIC positive patients more often fulfilled SEPSIS-2 criteria for “coagulation abnormalities” (INR 1.5 or activated prothrombin time [aPTT] > 60 s) at sepsis onset [17].

**Table 1:** Study participant characteristics at sepsis onset in the HYPRESS trial

	SIC positive at sepsis onset	SIC positive only after sepsis onset	SIC negative during the entire course of the disease	*p* value SIC positive at onset vs. SIC negative
	Total (*n* = 45)	Total (*n* = 14)	Total (*n* = 208)	
Male sex—no. (%)	25/45 (55.6)	9/14 (64.3)	144/208 (69.2)	0.083
Age—years	69 [55–75]	69.5 [66.3–74.3]	68 [55–75]	0.830
Type of admission—no. (%)				0.002
Surgery (elective)	5/45 (11.1)	3/14 (21.4)	54/207 (26.1)	
Surgery (emergency)	5/45 (11.1)	3/14 (21.4)	50/207 (24.2)	
Non-surgery (elective)	0	0	8/207 (3.9)	
Non-surgery (emergency)	35/45 (77.8)	8/14 (57.1)	95 (45.9)	
SOFA	8 [7–10]	6 [4–9]	5 [4–7]	< 0.001
SOFA SIC score adapted	5 [4–7]	5.5 [3.8–7.3]	4 [3–6]	0.010
APACHE II	19.5 [16–27]	25 [15.5–28.8]	17 [14–21]	0.016
SAPS II	56 [47.5–64.5]	58 [47.8–73.8]	53 [46–59]	0.092
SAPS 3	65 [57–73.5]	64 [46–82]	57 [48.5–64.5]	< 0.001
Organ dysfunction—no. (%)				
CNS	16/45 (35.6)	4/14 (28.6)	46/206 (22.3)	0.085
Coagulation	28/45 (62.2)	2/14 (14.3)	11/208 (5.3)	< 0.001
Pulmonary	26/45 (57.8)	6/14 (42.9)	153/207 (73.9)	0.045
Renal	19/45 (42.2)	7/14 (50.0)	69/208 (33.2)	0.300
Microcirculatory	18/45 (40.0)	8/14 (42.9)	59/208 (28.4)	0.153

If not otherwise indicated, the data are presented as absolute frequencies and percentages in round brackets, or median and interquartile range [IQR]. Microcirculatory organ dysfunction defined in HYPRESS as: “Lactate  > 1.5 × above the normal upper range and/or base deficit  ≥ 5 mmol/l and/or metabolic acidosis with pH < 7.3 and/or depressed capillary refill/mottling and/or significant body edema (capillary leakage syndrome)” [14]

*APACHE* Acute Physiology and Chronic Health Evaluation, *CNS* central nervous system, *MV* minute ventilation, *SAPS* Simplified Acute Physiology Score, *SIC* sepsis-induced coagulopathy, *SIRS* systemic inflammatory response syndrome, *SOFA* Sequential (sepsis-related) Organ Failure Assessment

For a list of concomitant diseases, please see the Supplementary Appendix of the HYPRESS trial [14]

### Onset of SIC positivity

Of 222 patients who were SIC negative at sepsis onset, 14 patients (6.3%) developed SIC during the following days. Twelve of these 14 patients (85.7%) became SIC positive during the first 4 days following sepsis onset (Fig. 1).

### SIC and clinical outcomes of patients of the HYPRESS trial

**Fig. 2:**
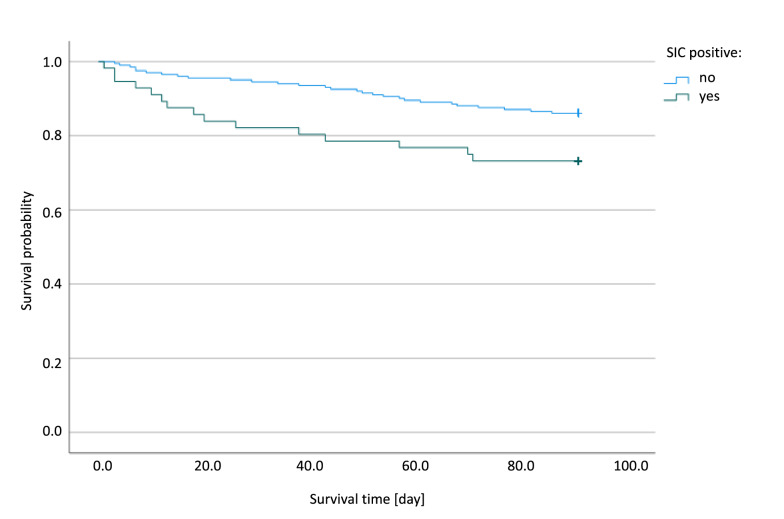
Kaplan–Meier plots of patients included in the HYPRESS trial [15]: 90-day survival (*p* = 0.014) Blue line: SIC negative patients. Green line: patients who were SIC positive at least once during the observation period. *SIC* sepsis-induced coagulopathy

Mortality rates (ICU, 28 day, 90 day, and 180 day) differed significantly between patients with and without SIC (Table 2 and Fig. 2). The positive predictive value of SIC (within the 14-d-observation period) to predict 180-day mortality was 37.5% (95%-CI 26.0–50.6%], whereas the negative predictive value was 81.7% [95%-CI 75.7–86.5%]. Logistic regression revealed a negative association of SIC with survival (*p* = 0.011; Additional file 1: Table S2). Of note, there was no difference regarding the necessity of mechanical ventilation between SIC positive and SIC negative patients with sepsis. However, SIC positive patients had a higher mean SOFA score until day 14 (*p* < 0.001) and needed renal replacement therapy (RRT) significantly more often until day 28 (*p* < 0.001; Table 2). Moreover, SIC was not associated with an increased ICU length of stay (ICU-LOS). There was no difference regarding gastro-intestinal bleeding events (defined as an acute bleeding which required transfusion of more than one unit of red blood cells within 24 h [14]) between SIC positive and SIC negative patients (Table 2).

**Table 2:** Outcome data of the secondary analysis of the HYPRESS trial [14]

	SIC (*n* = 59)	No SIC (*n* = 208)	*p* value
Septic shock or death within 14 days after randomization—no. (%)	21/59 (35.6)	38/208 (18.3)	0.007
28-day mortality—no. (%)	10/58 (17.2)	10/201 (5.0)	0.004
90-day mortality—no. (%)	15/56 (26.8)	28/201 (13.9)	0.027
180-day mortality—no. (%)	21/56 (37.5)	36/197 (18.3)	0.004
ICU mortality—no. (%)	10/58 (17.2)	9/203 (4.4)	0.003
Patients needing mechanical ventilation until day 28—no. (%)	33/58 (56.9)	109/203 (53.7)	0.765
Patients needing RRT until day 28—no. (%)	14/58 (24.1)	13/203 (6.4)	< 0.001
ICU length of stay—days [IQR]	8 [5.75–20.3]	8 [5–15]	0.438
Hospital length of stay—days [IQR]	26 [16–41.3]	25 [17–42]	0.797
Ventilator-free days in ICU—days [IQR]	5 [2–7]	4 [2–7]	0.815
RRT-free days in ICU until ICU discharge—days [IQR]	6 [4–15]	7 [4–12]	0.542
Mean SOFA score until day 14 [IQR]	7.67 [5–10.1]	4.35 [3.3–5.9]	< 0.001
Critical bleeding events	1/59 (1.7)	4/208 (1.9)	1.000

*ICU* intensive care unit, *IQR* interquartile range, *RRT* renal replacement therapy, *SIC* sepsis-induced coagulopathy, *SOFA* Sequential (sepsis-related) Organ Failure Assessment

Of the 45 patients who were SIC positive on admission to the ICU, 34 recovered from SIC during their stay on the ICU. In 11 patients SIC was persistent (defined as last documented values corresponding to a positive SIC score). Persistence of SIC was associated with a higher mortality (ICU, in-hospital, 14 day, 28 day, 90 day) as well as with a higher mean SOFA score until day 14 and a higher percentage of patients needing RRT until day 28 (Additional file 1: Table S3).

### PSSC prediction of SIC

An increased ISSC (> 0 points) at sepsis onset had a sensitivity of 93.2% and a specificity of 54.3% to predict SIC during the 14-day observation period (Additional file 1: Table S4). The positive predictive value (PPV) was 36.7%, the negative predictive value (NPV) 96.6%. In contrast, an increased PSSC reached a sensitivity of 84.8%, a specificity of 83.7%, a PPV of 59.5%, and a NPV of 95.1% Additional file 1: Tables S5 andS6.

### Estimating SIC prevalence and mortality using the PSSC in patients of the SISPCT trial

**Fig. 3:**
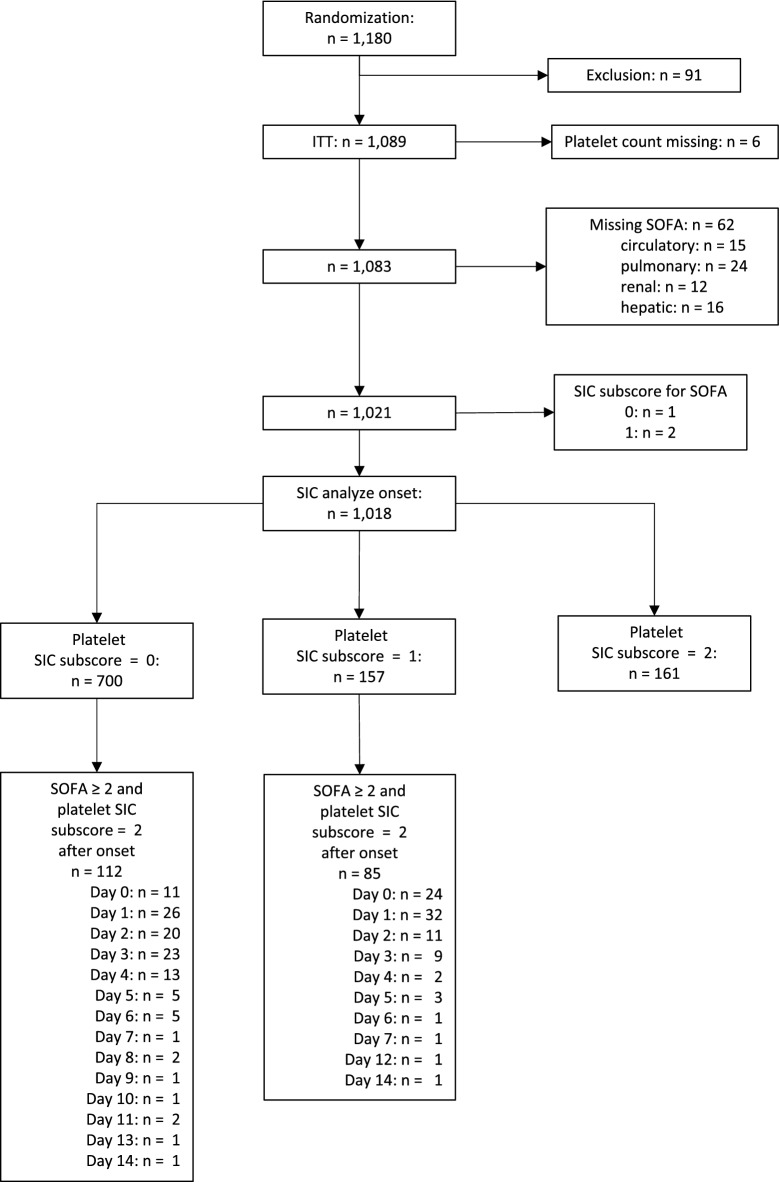
Flow diagram detailing the selection of patient groups analyzed from the SISPCT trial [15]. *ITT* intention to treat, *SIC* sepsis-induced coagulopathy; *SOFA* Sequential (sepsis-related) Organ Failure Assessment

**Fig. 4:**
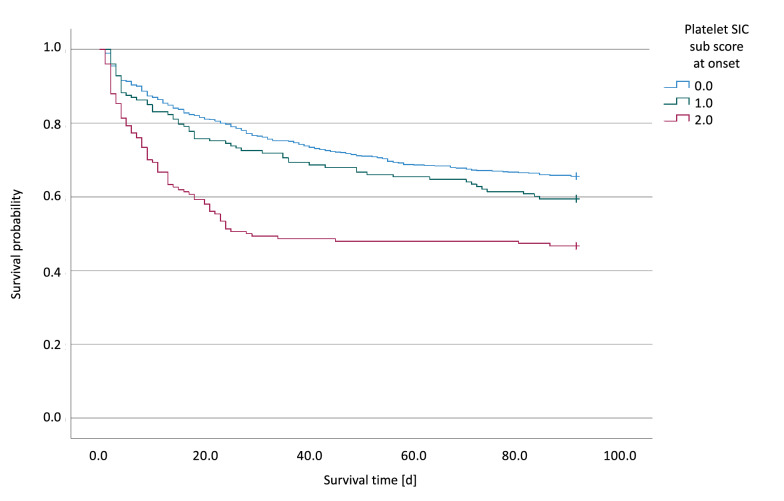
Kaplan–Meier plots of patients included in the SISPCT trial, stratified for the platelet-sepsis-induced coagulopathy subscore (PSSC) at sepsis onset. *N* = 675 patients having a PSSC of 0 (blue line), *n* = 153 patients having a score of 1 (green line), and *n* = 150 patients having a score of 2 (red line); *p* < 0.001

We analyzed 1,018 of 1,089 patients included in the SISPCT trial fulfilling SEPSIS-3 criteria (Fig. 3). At sepsis onset, 318 of 1018 (31.2%) patients had a PSSC of 1 or 2 (Fig. 3). Moreover, 112 of 700 (16.0%) patients having a sepsis-onset PSSC of 0 developed a PSSC of 2 (while having a SOFA score ≥ 2) during the 14-day observation period. In addition, 85 of 157 (54.1%) patients with PSSC of 1 at sepsis-onset developed a PSSC of 2 (while having a SOFA score ≥ 2) during the observation period. Using this modified SIC score (SOFA score ≥ 2 and PSSC = 2), the estimated SIC prevalence in the SISPCT trial was 24.2% (95% CI 21.6–26.9%). Additional file 1: Table S7 details the characteristics of patients having a PSSC of 0, 1, or 2. The PSSC was independent of the SISPCT intervention arms. There were no differences regarding sex or age. Like SIC scores in the HYPRESS trial, a higher PSSC in the SISPCT trial was associated with a higher SOFA score (PSSC 0: SOFA 9 [IQR 7–11]; PSSC 1: SOFA 11 [IQR 9–13]; PSSC 2: 13 [IQR 11–16]). In addition, a higher PSSC was associated with significantly higher 90-day mortality (PSSC 0: 34.5% [95%-CI 30.9–38.0%] vs. PSSC 1: 40.5% [95%-CI 33.1–48.4%] and PSSC 2: 53.3% [95%-CI 45.4–61.1%]; *p* < 0.001) (Fig. 4).

## Discussion

In this secondary analysis of the HYPRESS and SISPCT trials conducted in German ICUs, we observed a significantly lower SIC prevalence (16.9% at sepsis onset and 22.1% during the observation period) in patients with sepsis but not septic shock (at the time of inclusion) and 24.2% in patients with septic shock than had been reported in previous studies from Asia (40–60%) [4, 10, 14, 21]. Furthermore, SIC was either already present at sepsis onset or developed within the first 4 days. This observation is in line with the pathophysiology of SIC being most likely triggered by a dysregulated interaction between the innate immune response and the coagulation system [22]. To address these special features of SIC and to differentiate SIC from other DIC subtypes, Iba and colleagues introduced the SIC score [4, 21]. By excluding the parameters D-dimer and fibrinogen, the SIC score is more likely to detect sepsis-induced coagulopathy at an earlier stage than previous screening tools [23]. However, this also means that not all SIC positive patients suffer from overt DIC. The SIC score was developed “based on the results of logistic regression analyses” using data from 1,498 patients [4]. However, the included patients were highly preselected, since all patients suffered from “DIC according to the Japanese Ministry Health and Labor Welfare Diagnostic Criteria for DIC” and were treated with thrombomodulin alpha [4, 11]. In consequence, SIC prevalence was  > 60%. [4]. Therefore, it is not surprising that the observed prevalences in our study are lower although the median SIC-adapted SOFA scores (at the time of inclusion) of these patients were comparable to those of the patients analyzed from the HYPRESS trial (5 [IQR 3–7] vs. 5 [IQR 4–7]) [4]. However, there are several more studies reporting high SIC prevalences. Another study from Japan reported a SIC prevalence of 61.4% [5], and Ding and colleagues [5, 24] even found a SIC prevalence of 67.9% in a Chinese cohort of SEPSIS-3 patients. The most recent validation of the SIC sore is from 2021 [10]. In their secondary analysis, Tanaka and colleagues included only patients fulfilling sepsis criteria according to SEPSIS-3 [10]. The reported SIC incidences were 42.2% in patients not needing vasopressor therapy during their ICU stay and 66.4% in patients requiring vasopressors [10]. In the only European study that calculated SIC prevalence in patients with sepsis and vasopressor requirements, Julie Helms and colleagues reported a positive SIC Score in even 84.2% of the cases [25]. However, the SIC prevalence could have been overestimated in the latter study, since the additional condition introduced by Iba and colleagues (PSSC + ISSC ≥ 3) was not taken into account by Helms et al. when calculating the SIC score [4, 25]. In summary, the prevalence of SIC seems to vary significantly not only depending on the sepsis definition used, but also depending on the composition of the underlying cohort. As the HYPRESS trial included patients with severe sepsis but not shock with only about one fifth of the patients developing septic shock during the observation period (median SOFA Score = 5) and the SISPCT trial included patients with sepsis and septic shock with a median SOFA score of 10 (including 86.7% of patients with shock [15]), our secondary analysis includes two cohorts with different disease severities. However, in both trials SIC prevalence is considerably lower than previously reported. This is surprising as most of the prevalence studies mentioned above referred to patients with severe sepsis and septic shock according to the SEPSIS-2 definition and the patient groups covered by the definitions of “severe sepsis” (according to SEPSIS-2) and “sepsis” (according to SEPSIS-3) overlap widely [26]. Against this background, it is remarkable that the prevalence observed in both of our cohorts are lower by a factor of 2–3.

In this context, it is striking that almost all of the studies on the SIC score mentioned (even the one published in 2021) included patients that were treated for sepsis between 2011 and 2014 [4, 5, 10, 24]. At the same time, it is important to know that the recommendation for routine VTE prophylaxis was not included in the Japanese Sepsis Guideline before 2016 [13, 27]. The chapter did not exist in the 2014 version [27]. It was not until 2012 that the SCC recommended routine VTE prophylaxis for patients with sepsis for the first time [28]. There is no published data on how and whether pharmaceutical VTE prophylaxis was administered during this period. It can be assumed that it was heterogeneous and this fact could have had an impact on the SIC prevalence.

At the same time, mortality of SIC positive patients in our study was comparable to previous reports. SIC was associated with a 90-day mortality between 26.8% (HYPRESS) and 53.3% (SISPCT). This is comparable to the 28-day-mortality in SIC positive patients reported by Iba and collegues ranging between 30% in patients with a SIC score of 4–45% in patients with a SIC score of 6 [4]. Tanaka and colleagues also observed increased in-hospital-mortalities in SIC positive patients (requiring vasopressors: 35.8% vs. 27.9%; not requiring vasopressors: 15.6% vs. 12.2%) [10].

The fact that mortality of SIC positive patients in our observation was comparable to those of SIC positive patients in previous reports is remarkable knowing that the SSC guidelines and the Japanese Clinical Practice Guidelines for Management of Sepsis and Septic Shock differ substantially with regard to their recommendations for anticoagulation [13, 29]. While both the German guidelines and the SSC guidelines recommend pharmaceutical prophylaxis of venous thromboembolism (VTE) with unfractionated heparin or low molecular weight heparins, they do not provide any recommendation regarding the treatment of SIC or overt DIC [29, 30]. In contrast, the Japanese guidelines recommend screening for DIC and the replacement of antithrombin as well as the administration of thrombomodulin alpha in case of SIC [12]. Moreover, older versions of the Japanese guidelines for the management of sepsis suggested the use of protease inhibitors, or heparinoids at doses exceeding VTE prophylaxis [27]. Only since 2016 there has been a recommendation against the use of heparin as standard treatment for patients with sepsis and DIC and against the use of protease inhibitors [13]. As a result, about 50% of patients included in the major Japanese studies received at least one of the following medications: antithrombin, thrombomodulin alpha, protease inhibitors, or heparinoids at doses exceeding VTE prophylaxis [10]. In contrast, most patients in the HYPRESS and SISPCT trials received only pharmacological VTE prophylaxis, most likely because the German sepsis guidelines recommend against the use of antithrombin due to low evidence and augmented risk for severe bleeding events [30].

Our work has several strengths as well as limitations. Using two well-characterized cohorts of patients, which were included in two German multicenter RCTs within the SepNet Critical Care Trials network, strengthens the internal validity of our study. In both trials, patients were treated at more than 30 study sites, supporting the generalizability of our findings to other health care settings respecting the SSC guidelines. This is the first work to evaluate the performance of the SIC score in a population of sepsis patients receiving anticoagulation and prophylaxis of venous thromboembolism (VTE) according to the International Guidelines for Management of Sepsis and Septic Shock by the SSC [13, 16, 17]. Moreover, this is the first work that has been able to discuss the onset of SIC, because detailed daily data during the 14-day observation period were available.

One limitation is that cases of “late-onset” SIC with an onset after 14 days were not captured. However, our data highlight SIC rather as a complication of early sepsis, making the first 4 days after sepsis diagnosis the most important. Considering the described crosstalk between the innate immune response and the coagulation system, it seems reasonable that patients becoming SIC positive after 14 days might have had either a second infection or another medical condition (e.g. severe bleeding complication), which are accompanied by a drop in platelet count or a rise in INR. Moreover, such a hypothetical “late-onset” case of SIC after day 14 would have been without a major clinical impact as the median ICU-LOS in SIC negative patients was 8 days with a range of [5–15]. Another limitation is that we were unable to calculate the SIC prevalence in the SISPCT trial, because INR data had not been collected. The SIC score requires two conditions to be met to be considered positive: First, the total SIC score has to be  ≥ 4 points, second, as an additional condition, the sum of PSSC and ISSC has to be  ≥ 3 points. To fulfill the second condition, SIC positive patients must have a PSSC > 0. By counting only patients who had a PSSC of 2 at onset (while having a SOFA score ≥ 2), we only counted patients with a high probability of having or developing SIC. On the one hand, we might have underestimated the SIC prevalence by missing some patients with a PSSC of 1 and an ISSC of 2 during the course of the disease. On the other hand, SIC prevalence was likely overestimated because not all patients with a PSSC of 2 necessarily have an ISSC > 0. However, as both effects balance each other to a certain extent, our estimation seems to be quite exact.

## Conclusion

With a prevalence of 22.1% (HYPRESS)—24.2% (SISPCT) and an association with mortality, SIC is relevant in patients with sepsis and septic shock, although the prevalence is lower than previously reported. If SIC occurred, it was either present at the time of sepsis diagnosis or occurred during the first 4 days following sepsis diagnosis. In comparison with the ISSC, the PSSC appears to be more specific in order to predict SIC during the course of the disease. SIC was associated with mortality in two study populations representing different sepsis severity. Moreover, SIC was associated with a higher morbidity and as its occurrence within the first days of hospitalization should be perceived as a warning sign.

## Supplementary Information

**Additional file 1: Table S1. **Comparison of scores measuring sepsis associated coagulopathy (adapted from Saito et al. [1], Iba et al. [2], and Schmoch et al. [3]). **Table S2. **Multivariate regression analysis 180 d mortality of HYPRESS patients. **Table S3. **Association of SIC persistence with mortality and morbidity. **Table S4. **Performance of the ISSC as test to predict SIC. **Table S5. **Performance of the PSSC as test to predict SIC. **Table S6 **Maximum platelet SIC subscore after onset grouped by platelet SIC subscore at onset. **Table S7. **Part 1: Sepsis onset characteristics of SISPCT patients [6] grouped by PSSC.

## Data Availability

Access to study data may be granted following a formal request to the Study Management Committee (Studienleitkommission) of the SepNet Critical Care Trials group for approval. It can be contacted at [14].

## Abbreviations

CI: Confidence interval
DIC: Disseminated intravascular coagulopathy
HYPRESS:Effect of Hydrocortisone on Development of Shock Among Patients With Severe Sepsis: The HYPRESS randomized clinical trial
INR: International normalized ratio
IQR: Interquartile range
ISTH: International Society on Thrombosis and Haemostasias
ISSC: INR sepsis induced coagulopathy subscore
ITT: Intention to treat
JAAM: Japanese Association for Acute Medicine
PSSC: Platelet sepsis-induced coagulopathy subscore
PPV: Positive predictive value
RCT: Randomized controlled trial
SD: Standard derivation
SepNet: German sepsis competence network
SIC: Sepsis-induced coagulopathy
SISPCT: Effect of sodium selenite administration and procalcitonin-guided therapy on mortality in patients with severe sepsis or septic shock
SOFA: Sequential (sepsis-related) organ failure assessment
SSC: Surviving sepsis campaign
VTE: Venous thromboembolism
