# Supplementary material for: The prevalence of sepsis-induced coagulopathy in patients with sepsis – a secondary analysis of two German multicenter randomized controlled trials
Source: Ann Intensive Care. 2023 Jan 12;13:3. doi: 10.1186/s13613-022-01093-7 (PMC9837358; doi:10.1186/s13613-022-01093-7)
Supplement: Supplementary file 1 — Additional file 1: Table S1. Comparison of scores measuring sepsis associated coagulopathy (adapted from Saito et al. [1], Iba et al. [2], and Schmoch et al. [3]). Table S2. Multivariate regression analysis 180 d mortality of HYPRESS patients. Table S3. Association of SIC persistence with mortality and morbidity. Table S4. Performance of the ISSC as test to predict SIC. Table S5. Performance of the PSSC as test to predict SIC. Table S6 Maximum platelet SIC subscore after onset grouped by platelet SIC subscore at onset. Table S7. Part 1: Sepsis onset characteristics of SISPCT patients [6] grouped by PSSC. [file 13613_2022_1093_MOESM1_ESM.pdf]

Additional file to Schmoch et al. The prevalence of sepsis-induced coagulopathy in patients with sepsis – a secondary analysis of two German multicenter randomized controlled trials

Table S1. Comparison of scores measuring sepsis associated coagulopathy (adapted from Saito et al. (1), Iba et al.(2), and Schmoch et al.(3))

	**ISTH score** (4)	**JAAM score** (5)	**SIC score** (2)	**Points**
Underlying medical condition that makes DIC likely	Required	Required	Not required	
SIRS score	-	0–2		0
		≥3		1
Platelet count	> 100/nL	≥ 120/nL	> 150	0
	≤ 100/nL	80–120/nL<br>(or decrease > 30%/24 h)	> 100 and < 150/nL	1
	≤ 50/nL			2
		<80/nL<br>(or decrease > 50%/24 h)		3
Prothrombin time (PT-ratio)	< 3 s	(< 1.2)		0
	3–6 s	(≥ 1.2)	(≥ 1.2 and < 1.4)	1
	≥ 6 s		(≥ 1.4)	2
Fibrinogen	> 100 mg/dL	-	-	0
	-	-	-	1
	≤ 100 mg/dL	-	-	2
FDP/D-Dimer	Normal	< 10 µg/mL	-	0
		10–25 µg/mL	-	1
	Slightly increased		-	2
	Significantly increased	≥ 25 µg/mL	-	3
SOFA score			1	1
			≥ 2	2
Total DIC score	< 5 Points	< 4 Points	< 4 Points	No DIC/SIC
	≥ 5 Points	≥ 4 Points	≥ 4 Points<br>(with PT-INR plus Platelet count exceeding 2)	DIC/SIC

ISTH: International Society on Thrombosis and Haemostasis; JAAM: Japanese Association for Acute Medicine; DIC: disseminated intravascular coagulation; SIRS: systemic inflammatory response syndrome; SIC: sepsis-induced coagulopathy; SOFA: Sequential (sepsis-related) Organ Failure Assessment

Table S2. Multivariate regression analysis 180 d mortality of HYPRESS patients

Predictive Variable	Reference Category	OR	95% CI	p-value
Age [y]		1.04	1.01-1.06	0.007
Gender				
female	male	0.91	0.46-1.80	0.789
Treatment Arm				
Hydrocortisone	Placebo	1.66	0.89-3.12	0.114
SOFA (adapted for SIC)		1.20	1.03-.1.40	0.018
SIC				
yes	no	2.46	1.23-4.95	0.011

Table S3. Association of SIC persistence with mortality and morbidity

	**SIC resolved (n = 34)**	**SIC sustained (n = 11)**	***p* value**
14-day mortality – no. (%)	1/33 (3.0) [0.05-15.3]	4/11 (36.4) [15.2-64.6]	0.010
28-day mortality – no. (%)	1/33 (3.0) [0.05-15.3]	7/11 (63.6) [35.4-84.8]	<0.001
90-day mortality – no. (%)	5/31 (16.1) [7.1-32.6]	7/11 (63.6) [35.4-84.8]	0.006
180-day mortality – no. (%)	10/31 (32.3) [18.6-49.9]	7/11 (63.6) [35.4-84.8]	0.086
ICU mortality – no. (%)	2/33 (6.1) [1.7-19.6]	6/11 (54.5) [28.0-78.7]	0.001
Hospital mortality – no. (%)	2/33 (6.1) [1.7-19.6]	6/11 (54.5) [28.0-78.7]	0.001
Patients needing RRT until day 28 – no. (%)	3/33 (9.1) [3.1-23.6]	6/11 (54.5) [28.0-78.7]	0.004
ICU length of stay – days [IQR]	6 [5-21]	14 [7-18]	0.524
Mean SOFA score until day 14 [IQR]	6.25 [4.5-8.17]	10.89 [8.38–11.99]	0.001

IQR = interquartile range; SIC: sepsis-induced coagulopathy

Table S4. Performance of the ISSC as test to predict SIC

INR SIC subscore at sepsis onset	SIC during observation period	No SIC during observation period	Total
1 or 2 points	55	95	**150**
0 points	4	113	**117**
**Total**	**59**	**208**	**267**

Sensitivity: 55/59 = 93.2% [95%-CI: 83.8%-97.3%]
Specificity: 113/208 = 54.3% [95%-CI: 47.5%-61.0%]
False positive: 95/150 = 63.3% [95%-CI: 55.4%-70.6%]
False negative: 4/117 = 3.4% [95%-CI: 1.3%-8.5%]
Positive predictive value: 55/150 = 36.7% [95%-CI: 29.4%-44.6%]
Negative predictive value: 113/117 = 96.6% [95%-CI: 91.5%-98.7%]
INR: international normalized ratio, ISSC: INR SIC subscore, SIC: sepsis-induced coagulopathy

Table S5. Performance of the PSSC as test to predict SIC

Platelet SIC subscore at sepsis onset	SIC during observation period	No SIC during observation period	Total
1 or 2 points	50	34	**84**
0 points	9	174	**183**
**Total**	**59**	**208**	**267**

Sensitivity: 50/59 = 84.8% [95%-CI: 73.5%-91.8%]
Specificity: 174/208 = 83.7% [95%-CI: 78.0%-88.1%]
False positive: 34/84 = 40.5% [95%-CI: 30.6%-51.2%]
False negative: 9/183 = 4.9% [95%-CI: 2.6%-9.1%]
Positive predictive value: 50/84 = 59.5% [95%-CI: 48.8%-69.4%]
Negative predictive value: 174/183 = 95.1% [95%-CI: 90.9%-97.4%]
SIC: sepsis-induced coagulopathy; PSSC: platelet SIC subscore

Table S6 Maximum platelet SIC subscore after onset grouped by platelet SIC subscore at onset

**Maximum platelet SIC subscore after onset**	**Platelet SIC subscore at sepsis onset**			**Total**
	1 – n (%)	2 – n (%)	3 – n (%)	
0	434 (62.4)	16 (10.2)	4 (2.5)	454 (45)
1	142 (20.4)	53 (33.8)	13 (8.3)	208 (20.6)
2	119 (17.1)	88 (56.1)	140 (89.2)	347 (34.4)
**Total**	695 (100)	157 (100)	157 (100)	1009 (100)

SIC: sepsis-induced coagulopathy

Table S7. Part 1: Sepsis onset characteristics of SISPCT patients (6) grouped by PSSC

Sepsis-onset PSSC	PSSC = 0 (n = 700)	PSSC = 1 (n = 157)	PSSC = 2 (n = 161)	Total (n = 1018)	P value
Treatment arm – no. (%)					0.942
SelPCT	178/700 (25.4)	36/157 (22.9)	43/161 (26.7)	257/1018 (25.2)	
SelKon	173/700 (24.7)	40/157 (25.5)	42/161 (26.1)	255/1018 (25.0)	
PlacPCT	179/700 (25.6)	38/157 (24.2)	41/161 (25.5)	258/1018 (25.3)	
PlacKon	170/700 (24.3)	43/157 (27.4)	35/161 (21.7)	248/1018 (24.4)	
Male sex – no. (%)	445/700 (63.6)	106/157 (67.5)	94/161 (58.4)	645/1018 (63.4)	0.235
Age – years	68 [58–76]	70 [59–75.5]	64 [54.5–73]	68 [57–75]	0.007
Type of admission – no. (%)					0.868
Surgery (elective)	82/700 (11.7)	15/157 (9.6)	19/161 (11.8)	116/1018 (11.4)	
Surgery (emergency)	317/700 (45.3)	78/157 (49.7)	73/161 (45.3)	468/1018 (46.0)	
Non-surgery (emergency)	301/700 (43.0)	64/157 (40.8)	69/161 (42.9)	434/1018 (42.6)	
SOFA	9 [7–11]	11 [9–13]	13 [11–16]	10 [8–12]	**< 0.001*** **
SOFA SIC score adapted	8 [7–9]	9 [7–10]	10 [8–11.5]	8 [7–10]	**< 0.001*** **
APACHE II	23 [19–28]	24 [19–30]	26 [21–32]	24 [19–29]	**< 0.001*** **
SAPS II	61 [54–70]	62 [54–75.25]	65 [58–75]	62 [55–71]	**0.003** **
SIRS criteria – no. (%)					
Temperature ≤ 36°C or ≥ 38°C	495/700 (70.7)	119/157 (75.8)	115/161 (71.4)	729/1018 (71.6)	0.442
Heart rate ≥ 90/min	628/700 (89.7)	143/157 (91.1)	153/161 (95.0)	924/1018 (90.8)	0.109
Tachypnea/hypocapnia/MV	674/700 (96.3)	153/156 (98.1)	158/161 (98.1)	985/1017 (96.9)	0.401
Leukocytosis/leukopenia/left shift	614/700 (87.7)	115/157 (73.2)	138/161 (85.7)	867/1018 (85.2)	**< 0.001*** **
Organ dysfunction – no. (%)					
CNS	126/686 (18.4)	28/154 (18.2)	37/157 (23.6)	191/997 (19.2)	0.310
Coagulation	20/699 (2.9)	25/156 (16.0)	136/157 (86.8)	181/1012 (17.9)	**< 0.001*** **
Pulmonary	497/698 (71.2)	110/157 (70.1)	126/160 (78.8)	733/1015 (72.2)	0.127
Septic shock	607/700 (86.7)	138/157 (87.9)	145/161 (90.1)	890/1018 (87.4)	0.504
Renal	308/699 (44.1)	87/157 (55.4)	100/161 (62.1)	495/1017 (48.7)	**< 0.001*** **
Metabolic	381/700 (54.4)	108/157 (68.8)	113/161 (70.2)	602/1018 (59.1)	**< 0.001*** **

If not otherwise indicated, the data are presented as absolute frequencies and percentages in brackets, or the median and interquartile range
PSSC: platelet sepsis-induced coagulopathy subscore; SOFA: Sequential (sepsis-related) Organ Failure Assessment; SIC: sepsis-induced coagulopathy; APACHE: Acute Physiology and Chronic Health Evaluation, PlacKon = patients randomized to placebo treatment and conventional therapy guiding in the SISPCT trial (6) ; PlacPCT = patients randomized to placebo treatment and procalcitonin guided therapy in the SISPCT trial (6); SAPS: Simplified Acute Physiology Score; SelKon = patients randomized to sodium selenite treatment and conventional therapy guiding in the SISPCT trial (6); SelPCT = patients randomized to sodium selenite treatment and procalcitonin guided therapy in the SISPCT trial (6); SIRS: systemic inflammatory response syndrome; CNS: central nervous system; ICU: intensive care unit. For a list of concomitant diseases, please see the Supplementary Appendix of the original publication (6)

Table S7 Part 2: Sepsis onset characteristics of SISPCT patients (6) grouped by PSSC

**Sepsis-onset PSSC**	**PSSC = 0** (n = 700)	**PSSC = 1** (n = 157)	**PSSC = 2** (n = 161)	**Total** (n = 1018)	**P value**
Source of infection – no. (%)					0.014
Community	297/700 (42.4)	66/157 (42.0)	83/161 (51.6)	446/1018 (43.8)	
Nosocomial (ICU)	158/700 (22.6)	49/157 (31.2)	38/161 (23.6)	245/1018 (24.1)	
Nosocomial (Ward)	245/700 (35.0)	42/157 (26.8)	40/161 (24.8)	327/1018 (32.1)	
Focus of primary infection					
Known focus – no. (%)	680/700 (97.1)	148/157 (94.3)	154/161 (95.7)	982/1018 (96.5)	0.176
Pneumonia[#]	295/680 (43.4)	69/148 (46.6)	66/154 (42.9)	430/982 (43.8)	0.747
Respiratory tract (other)	28/680 (4.1)	9/148 (6.1)	6/154 (3.9)	43/982 (4.4)	0.543
Thoracic	34/680 (5.0)	4/148 (2.7)	3/154 (1.9)	41/982 (4.2)	0.145
Gastrointestinal	54/680 (7.9)	13/148 (8.8)	11/154 (7.1)	78/982 (7.9)	0.870
Intraabdominal	266/680 (39.1)	49/148 (33.1)	42/154 (27.3)	357/982 (36.4)	0.015
Primary bacteremia	18/680 (2.6)	4/148 (2.7)	10/154 (6.5)	32/982 (3.3)	0.062
Bones /soft tissue	54/680 (7.9)	6/148 (4.1)	20/154 (13.0)	80/982 (8.1)	**0.017***
Surgical wound	31/680 (4.6)	2/148 (1.4)	5/154 (3.2)	38/982 (3.9)	0.169
Urogenital	57/680 (8.4)	14/148 (9.5)	18/154 (11.7)	89/982 (9.1)	0.428
Catheter	18/680 (2.6)	5/148 (3.4)	5/154 (3.2)	28/982 (29)	0.728
CNS	0/680	2/148 (1.4)	1/154 (0.6)	3/982 (0.3)	**0.020***
Cardiovascular	3/680 (0.4)	1/148 (0.7)	1/154 (0.6)	5/982 (0.5)	0.490

The data are presented as absolute frequencies and percentages in brackets
PSSC: platelet sepsis-induced coagulopathy subscore; CNS: central nervous system
For a list of concomitant diseases, please see the Supplementary Appendix

References

1. Saito S, Uchino S, Hayakawa M, Yamakawa K, Kudo D, Iizuka Y, u. a. Epidemiology of disseminated intravascular coagulation in sepsis and validation of scoring systems. J Crit Care. 1. April 2019;50:23–30.

2. Iba T, Nisio MD, Levy JH, Kitamura N, Thachil J. New criteria for sepsis-induced coagulopathy (SIC) following the revised sepsis definition: a retrospective analysis of a nationwide survey. BMJ Open. 27. September 2017;7(9):e017046.

3. Schmoch T, Brenner T, Becker-Pennrich A, Hinske LC, Weigand MA, Briegel J, u. a. Therapie der Sepsis-induzierten Koagulopathie. Ergebnisse einer deutschlandweiten Umfrage auf Intensivstationen. Anaesthesist. 2021;

4. Taylor FB, Toh CH, Hoots WK, Wada H, Levi M, Scientific Subcommittee on Disseminated Intravascular Coagulation (DIC) of the International Society on Thrombosis and Haemostasis (ISTH). Towards definition, clinical and laboratory criteria, and a scoring system for disseminated intravascular coagulation. Thromb Haemost. November 2001;86(5):1327–30.

5. Gando S, Iba T, Eguchi Y, Ohtomo Y, Okamoto K, Koseki K, u. a. A multicenter, prospective validation of disseminated intravascular coagulation diagnostic criteria for critically ill patients: comparing current criteria. Crit Care Med. März 2006;34(3):625–31.

6. Bloos F, Trips E, Nierhaus A, Briegel J, Heyland DK, Jaschinski U, u. a. Effect of Sodium Selenite Administration and Procalcitonin-Guided Therapy on Mortality in Patients With Severe Sepsis or Septic Shock: A Randomized Clinical Trial. JAMA Intern Med. 1. September 2016;176(9):1266–76.
